# Identification of resistance of *Plasmodium falciparum* to artesunate-mefloquine combination in an area along the Thai-Myanmar border: integration of clinico-parasitological response, systemic drug exposure, and *in vitro* parasite sensitivity

**DOI:** 10.1186/1475-2875-12-263

**Published:** 2013-07-30

**Authors:** Kesara Na-Bangchang, Phunuch Muhamad, Ronnatrai Ruaengweerayut, Wanna Chaijaroenkul, Juntra Karbwang

**Affiliations:** 1 International College of Medicine, Thammasat University, Klongluang, Pathumthanee, Thailand; 2 Mae Sot General Hospital, Mae Sot, Tak province, Thailand; 3 Department of Clinical Product Development, Institute of Tropical Medicine, Nagasaki University, Nagasaki, Japan

**Keywords:** *Plasmodium falciparum*, Drug resistance, Artesunate, Mefloquine, *pfmdr1* copy number, *in vitro* sensitivity

## Abstract

**Background:**

A markedly high failure rate of three-day artesunate-mefloquine was observed in the area along the Thai-Myanmar border.

**Methods:**

Identification of *Plasmodium falciparum* isolates with intrinsic resistance to each component of the artesunate-mefloquine combination was analysed with integrated information on clinico-parasitological response, together with systemic drug exposure (area under blood/plasma concentration-time curves (AUC)) of dihydroartemisinin and mefloquine, and *in vitro* sensitivity of *P. falciparum* in a total of 17 out of 29 *P. falciparum* isolates from patients with acute uncomplicated falciparum malaria. Analysis of the contribution of *in vitro* parasite sensitivity and systemic drug exposure and relationship with *pfmdr1* copy number in the group with sensitive response was performed in 21 of 69 cases.

**Results:**

Identification of resistance and/or reduced intrinsic parasitocidal activity of artesunate and/or mefloquine without pharmacokinetic or other host-related factors were confirmed in six cases: one with reduced sensitivity to artesunate alone, two with resistance to mefloquine alone, and three with reduced sensitivity to artesunate combined with resistance to mefloquine. Resistance and/or reduced intrinsic parasitocidal activity of mefloquine/artesunate, together with contribution of pharmacokinetic factor of mefloquine and/or artesunate were identified in seven cases: two with resistance to mefloquine alone, and five with resistance to mefloquine combined with reduced sensitivity to artesunate. Pharmacokinetic factor alone contributed to recrudescence in three cases, all of which had inadequate whole blood mefloquine levels (AUC_0-7days_). Other host-related factors contributed to recrudescence in one case. Amplification of *pfmdr1* (increasing of *pfmdr1* copy number) is a related molecular marker of artesunate-mefloquine resistance and seems to be a suitable molecular marker to predict occurrence of recrudescence.

**Conclusions:**

Despite the evidence of a low level of a decline in sensitivity of *P. falciparum* isolates to artemisinins in areas along the Thai-Myanmar border, artemisinin-based combination therapy (ACT) would be expected to remain the key anti-malarial drug for treatment of multidrug resistance *P. falciparum*. Continued monitoring and active surveillance of clinical efficacy of ACT, including identification of true artemisinin resistant parasites, is required for appropriate implementation of malaria control policy in this area.

## Background

The emergence and spread of multidrug resistant *Plasmodium falciparum* is the key factor contributing to complexity in malaria control. To deal with the threat of resistance, artemisinin-based combination therapy (ACT) is being promoted as a strategy to conteract the increasing resistance of the parasite as well as to prevent disease transmission [1]. Despite the precautionary measures however, artemisinin-resistant *P. falciparum* malaria has emerged in western Cambodia and the bordering regions with Thailand, the hotspot of multidrug resistance parasites [2-10], and appears to be emerging in the western border of Thailand [11,12].

In Thailand, a three-day, artesunate-mefloquine combination regimen has been used as the first-line treatment for acute uncomplicated falciparum malaria throughout the country [13]. In a previous study which aimed at monitoring clinical efficacy of this three-day artesunate-mefloquine combination regimen during the year 2009 in 134 patients with acute uncomplicated falciparum malaria in the area along the Thai-Myanmar border, a markedly high failure rate was observed [11]. The 28- and 42-day cure rates calculated by Kaplan-Meier survival analysis with PCR correction for re-infection were 74.7 and 68.1%, respectively. It is noted that re-appearance of parasitaemia occurred as early as seven days after the first dose. In addition, there was a small but significant delay of parasite clearance in the group with recrudescence response (median (range) parasite clearance time 32.0 (28.0-34.0) h) compared with the sensitive group (26.0 (24.0-26.0) h). Only six (17.6%) and seven (20.5%) patients with recrudescence response, respectively, had parasitaemia and fever cleared within 24 hours. This observation is alarming and is of great concern if resistance of *P. falciparum* has actually developed and spread in this area. In the present study, identification of resistance/reduced sensitivity of *P. falciparum* in this border area to each component of this three-day, artesunate-mefloquine combination regimen was proposed based on clinico-parasitological response, with confirmed adequacy of anti-malarial systemic drug exposure during acute phase infection, and *in vitro* sensitivity of *P. falciparum* isolates to each combination partner. In addition, the possible link between the identified “resistance” cases and *P. falciparum* multidrug resistance 1 *(pfmdr1)* copy number, the candidate molecular markers of mefloquine and/or artesunate resistance was investigated.

## Methods

### Patients and study framework

**Figure 1:**
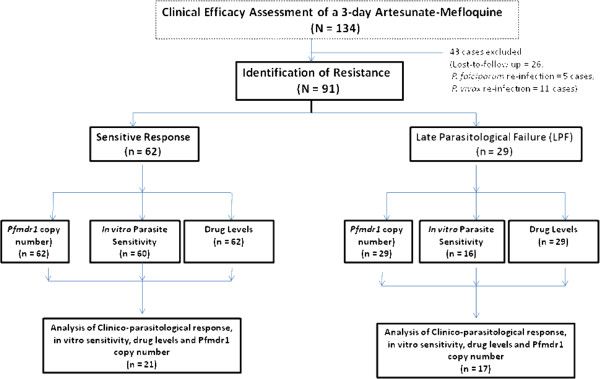
**Summary of cases included in the analysis of each step (clinico-parasitological response, *pfmdr1 *copy number, *in vitro *parasite sensitivity to artemisinin and mefloquine and drug levels (dihydroartemisinin and mefloquine). **^a^ = Reference [11].

The study was conducted at Mae Tao clinic for migrant workers, Tak Province, Thailand [11]. Figure 1 summarizes the total number of cases included in the study and number of cases included in each step of analysis. Prior to study, approval of the study protocol was obtained from the Ethics Committee of the Ministry of Public Health of Thailand. Written informed consents were obtained from all patients before study participation. The analysis for identification of resistance of *P. falciparum* to artesunate-mefloquine combination was performed in a total of 91 (62 cases with sensitive response and 29 with PCR-confirmed recrudescence) Burmese patients (47 males and 44 females, aged between 16 and 57 years) with acute uncomplicated *P. falciparum* malaria (median (95% CI) admission parasitaemia 5,512 (5,040-6,930)/μl] [14]). Re-appearance of parasitaemia in the 29 late parasitological failure (LPF) cases occurred between day 7 and 42, with significant prolongation of parasite and fever clearance time (PCT nd FCT) in patients with recrudescence compared with sensitive response (median (95% CI) 32.0 (28.0-34.0) *vs* 26.0 (24.0-26.0) h, and 32.0 (30.0-34.0) *vs* 26.0 (24.0-26.0) h, respectively). The study procedures and results of clinical efficacy assessment, including relationship with drug concentrations, were previously described in detail [11]. In brief, patients were treated with the standard three-day combination regimen of artesunate and mefloquine with primaquine (4 mg/kg body weight artesunate daily for three days; 750 and 500 mg mefloquine on the first and second day, respectively; 0.6 mg/kg body weight primaquine on the third day). All were admitted to the clinic during the course of treatment or until signs and symptoms of malaria disappeared. Prior to treatment, blood sample (5 ml) was collected from each patient for *in vitro* sensitivity testing of *P. falciparum* isolates to artesunate and mefloquine, genetic analysis (molecular markers of recrudescence and drug resistance) and determination of baseline anti-malarial drug concentrations (mefloquine, artesunate and its active plasma metabolite, dihydroartemisinin).

Patients were requested to return for follow-up on days 7, 14, 21, 28 and 42, or at any time if fever or symptoms suggestive of malaria developed. At each visit, a parasite count was performed (Giemsa-stain), and a detailed questionnaire for general symptoms was recorded. Blood samples were collected at specified time points for measurement of mefloquine (at one, two, six, 12, 24, 25, 36, 37, 48, 49 hours, and three and seven days after the first dose) and artesunate/dihydroartemisinin (at one, six, 12 and 24 hours after the first dose) concentrations. Malaria blood smears were obtained on enrolment and thereafter, twice daily until two consecutive slides were confirmed to be negative, as well as at every follow-up visit. Thick films were screened for 200 oil-immersion fields before declaring a slide negative. Asexual parasites and gametocytes were separately counted against 200 white blood cells (WBCs); if the parasite density was too numerous to count on the thick film, the number of parasites per 2,000 red blood cells (RBCs) on the thin film was counted. Parasite slope half-life for each patient was calculated from log_e_ (2)/k = 0.693, where k is the clearance rate constant [15].

*Plasmodium falciparum* genotyping of the three polymorphic genes for surface antigen 1 (*msp1*), merozoite surface antigen 2 (*msp2*), and glutamate-rich protein (*glurp*) was performed in paired samples collected prior to treatment and at the time of parasite re-appearance to distinguish between re-infection and recrudescence [16-18]. Blood sample (5 ml) was also collected from each individual with re-appearance of parasitaemia for determination of drug concentrations, *in vitro* sensitivity test, and evaluation of potential molecular marker of resistance, *P. falciparum* multidrug resistance 1 (*pfmdr1*) copy number. Clinical efficacy of the three-day course of artesunate-mefloquine was evaluated in the group of patients who completed the 42-day follow-up period. The classification of the therapeutic outcome was according to the WHO protocol [14]. Plasma concentrations of artesunate and dihydroartemisinin were measured using liquid chromatography mass-spectrometry (LC/MS) according to the method of Thuy and colleagues [19]. Determination of whole blood concentrations of mefloquine was performed using high performance liquid chromatography with UV-detection (HPLC-UV) according to the method developed by Karbwang and colleagues with modification [20].

### Assessment of *in vitro* sensitivity of *Plasmodium falciparum* isolates

The *in vitro* sensitivity test was accomplished in a total of 76 *P. falciparum* isolates (60, and 16 isolates collected pretreatment and at the time of recrudescence, respectively). *Plasmodium falciparum* 3D7 (chloroquine sensitive) and K1 (chloroquine resistant) were used as control *P. falciparum* clones. All were cultured according to the method of Trager and Jensen with modification [21]. *In vitro* sensitivity testing was performed in 96-flat-bottom wells sterile microtiter plate (Costar™, Corning, Massachusetts, USA) according to the methods of Rieckmann and colleagues [22]. Evaluation of sensitivity of *P. falciparum* isolates to mefloquine and artesunate was performed based on SYBR Green I assay [23]. The triplicate results of fluorescence intensity of each drug were calculated to obtain mean value. The dose response curves obtained from *in vitro* sensitivity assay were analysed by non-linear regression analysis using CalcuSyn™ software (Biosoft, Cambridge, UK). Results were expressed as IC_50_ value, which is defined as the concentration of anti-malarial drug that produces 50% inhibition of parasite development as compared to the control.

The criteria for discrimination between the resistant and sensitive parasite isolates to mefloquine was as follows: sensitive (IC_50_ ≤24 nM), and resistant (IC_50_ >24 nM) [24,25]. For artesunate, as there has been no clear cut-off level for artemisinin resistance, two criteria were applied. Susceptibility was classified into two levels according to the criteria of Pradines and colleagues [26], i e, sensitive (IC_50_ ≤10.5 nM), and resistant (IC_50_ >10.5 nM). In addition, IC_50_ value of greater than the upper limit of 95% CI of median defined from sensitive isolate group as previously applied [27], i e, IC_50_ >2.8 nM, was considered as declined sensitivity to artesunate [27].

### Determination of *pfmdr1* copy number by SYBR Green I quantitative real-time PCR (qRT-PCR)

Investigation of *pfmdr1* copy number was performed in a total of 120 *P. falciparum* isolates. Sixty-two samples were obtained from patients with sensitive response during the 42-day follow-up period. Twenty-nine paired samples (58 isolates) were obtained from patients with recrudescence before treatment and at the time of parasite reappearance. Genomic DNA was extracted from all samples using chelex resin modified technique [18]. SYBR Green I qRT-PCR was performed using iCycler™ real-time PCR machine (Bio-Rad, California, USA) according to the method described by Ferreira and colleagues with modification [28]. 3D7 (1 gene copy number) and Dd2 (4 gene copy number) *P. falciparum* were used as positive control clones for *pfmdr1* copy number analysis (provided by Professor Steven A Ward, Liverpool School of Tropical Medicine, UK). Distilled water was used as negative control.

### Data analysis

A total of 38 patients with acute uncomplicated *P. falciparum* malaria were included in the analysis. Identification of parasite isolates with intrinsic resistance/reduced sensitivity to mefloquine and/or artesunate was analysed in 17 of 29 cases with complete information on clinico-parasitological response, drug concentrations, and *in vitro* sensitivity. Analysis of the contribution of these three factors in the group with sensitive response was performed in 21 of 62 cases. Quantitative variables are summarized as median (95% confidence interval: 95% CI) values, and qualitative variables are presented as number (%) values. Three criteria were applied for identification of *P. falciparum* cases with intrinsic resistance to mefloquine and/or artesunate: (i) PCR-confirmed recrudescence during a 42-day follow-up with delayed parasite slope half-life; (ii) adequacy of mefloquine and/or dihydroartemisinin systemic drug exposure (AUC); and, (iii) *in vitro* parasite resistance/reduced sensitivity to mefloquine and/or artesunate as defined by the above criteria.

The upper limit of 95% CI of the parasite slope half-life in the group with sensitive response (2.99 h) was used as the cut-off for delayed parasite slope half-life. The lower limits of 95% CI of area under whole blood mefloquine conentration-time curve from day 0 to day 7 (AUC_0-7 days_) in patients with sensitive response (8.48 mg.day/ml) was used as a criterion for the adequacy of mefloquine systemic drug systemic drug exposure. The lower limit of 95% CI of plasma dihydroartemisinin concentration-time curve from hour 0 to hour 24 (AUC_0-24 h_) in patients with sensitive response (462 ng.h/ml) was used as a criterion for the adequacy of dihydroartemisinin systemic drug exposure. AUC was calculated using trapezoidal rule. Association between mefloquine and dihydroartemisinin systemic exposure (AUC_0-7 days_ and AUC_0-24h_) and treatment response was performed using Fisher's exact test.

Analysis of the correlation between IC_50_ values of mefloquine and artesunate, IC_50_ values of both drugs and *pfmdr1* copy number, as well as *pfmdr1* copy number and PCT was performed using Spearman’s (*rho*) correlation test at a statistical significance of *α* = 0.05 (SPSS version 15; SPSS, Chicago, Illinois, USA).

## Results

### Pretreatment *in vitro* sensitivity of *Plasmodium falciparum* isolates

The IC_50_ values of mefloquine in all isolates collected before treatment ranged from 6.0-118.4 nM, with median (95% CI) IC_50_ of 28.0 (21.2-41.0) nM. Based on the defined criteria, a total of 26 (43.3%) isolates were classified as mefloquine sensitive (median (95% CI) IC_50_ = 16.6 (12.9-19.7) nM). The remaining 34 (56.7%) isolates were classified as mefloquine resistant isolates (median (95%) of IC_50_ = 32.0 (35.0-62.2) nM).

The IC_50_ values of artesunate in all isolates collected before treatment ranged from 0.3-6.1 nM, with median (95% CI) IC_50_ of 1.9 (1.6-2.4) nM. Based on the criteria defined by Pradines and colleagues [26], all were considered sensitive to artesunate (median (95% CI) IC_50_ = 1.9 (1.6-2.4) nM). However, when upper limit of 95% CI of IC_50_ in the sensitive was applied as the cut-off criteria, declining in sensitivity to artesunate was observed in 22 (36.7%) isolates. It was noted however for the relatively wide variation of IC_50_ values ranging from 0.3 to 6.1 nM in all isolates.

A relatively strong positive significant correlation was observed between the IC_50_ values of mefloquine and artesunate (*r*^*2*^ = +0.701; *p* < 0.001).

### *Pfmdr1* copy number

**Figure 2:**
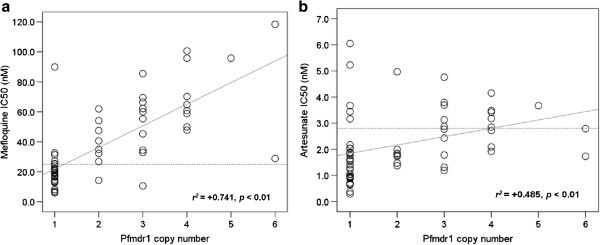
**Correlation between *pfmdr1 *copy number and *in vitro *sensitivity of *Plasmodium falciparum i*solates to (a) mefloquine, and (b) artesunate.** (Dashed lines represent the cut-off IC_50_ values for resistance to mefloquine/reduced sensitivity to artesunate).

The analysis of *pfmdr1* gene copy number was performed in a total 91 isolates (62 and 29 isolates from patients with sensitive response and recrudescence, respectively) recollected before treatment. Most isolates (40%) carried 1 gene copy, with markedly varied IC_50_ values. The median (95% CI) IC_50_ values of mefloquine in the isolates carrying one, two and three or more than three *pfmdr1* gene copies were 19.3 (14.6-21.5), 37.8 (14.3-61.9), and 60.8 (47.8-70.3) nM, respectively. The median (95% CI) IC_50_ values of artesunate in the isolates carrying one, two and three, or more than three *pfmdr1* gene copies, were 1.5 (1.0-1.9), 1.8 (1.4-5.0), and 2.9 (2.0-3.5) nM, respectively. A significant positive correlation was found between *pfmdr1* copy number and *in vitro* sensitivity of *P. falciparum* isolates to mefloquine (*r*^*2*^ = +0.741, *p* < 0.01), and artesunate (*r*^*2*^ = +0.485, *p* < 0.01) (Figures 2a and 2b).

### Identification of resistance of *Plasmodium falciparum* isolates to artesunate-mefloquine combination therapy and *pfmdr1* copy number as a candidate molecular marker of resistance

Identification of resistance of *P. falciparum* isolates to artesunate-mefloquine combination therapy was analysed in 17 out of 29 cases with confirmed recrudescence and with complete information on clinico-parasitological, drug concentrations, and *in vitro* sensitivity (Table 1). Based on the upper limit of 95% CI of the parasite slope half-life in patients with sensitive response (2.99 h), all of the patients with recrudescence had delayed parasite clearance rate. Recrudescence occurred between day 14 and 42. There was no significant difference in IC_50_ values of both mefloquine and artesunate in isolates collected before treatment from patients with sensitive and recrudescence responses (median (95% CI): 21.1 (14.6-34.5) *vs* 49.9 (19.4-69.5) nM (*p* = 0.057), and 1.8 (1.4-2.2) *vs* 2.8 (1.4-3.7) nM (*p* = 0.093) for mefloquine and artesunate, respectively). Analysis of paired isolates collected from patients with PCR-confirmed recrudescence (17 of 29 cases), before and at the time of parasite re-appearance, revealed no significant changes in sensitivity to both drugs. Median (95% CI) for IC_50_ of mefloquine in paired isolates before and at the time of recrudescence was 52.6 (19.4-70.2) and 54.9 (19.6-81.7) nM, respectively (*p* = 0.10). The corresponding values for mefloquine IC_50_ were 2.7 (1.2-3.7) and 3.4 (1.6-4.6) nM, respectively (*p* = 0.08).

**Table 1:** **Identification of resistance/reduced sensitivity of *P. falciparum *isolates to artesunate (AS)-mefloquine (MQ) combination therapy was analysed based on clinico-parasitological response, and *in vitro *sensitivity and systemic drug exposure during the acute phase of infection and relationship with *pfmdr1 *copy number in 17 cases with treatment failure following a three-day combination regimen**

**Systemic drug exposure**	**Case No.**	**Parasite slope half-life (h)**^**a**^	***In vitro *sensitivity (IC**_**50**_**: nM)**^**b**^		***Pfmdr1 *copy number**	**MQ/DHA Systemic exposure**		**Conclusion on cause(s) of treatment failure**
			**AS**	**MQ**		**AUC**_**0-7d**_**/ AUC**_**0-24h**_**/**	**Day R**^**e**^	
Adequate DHA exposure alone (n=5)	1	3.12	Reduced (3.5±0.42)	Resistant (58.8±5.1)	>1	7.55^c^	610 (day 21)	-Possible AS resistant
								-MQ resistant + Inadequate MQ exposure
								-Predicted by increased in *pfmdr1* copy number
	2	3.56	Reduced (4.8±1.07)	Resistant (69.5±4.7)	>1	8.02^c^	100 (day 28)	-Possible AS resistant
								-MQ resistant + Inadequate MQ exposure
								-Predicted by increased in *pfmdr1* copy number
	3	4.23	Sensitive (1.2±0.27)	Sensitive (10.6±1.9)	1	6.98^c^	300 (day 28)	-Inadequate MQ exposure
	4	3.88	Sensitive (0.7±0.13)	Sensitive (6.6±0.4)	1	7.99^c^	600 (day 33)	-Inadequate MQ exposure
	5	3.42	Sensitive (0.9±0.10)	Sensitive (19.4±0.7)	>1	8.07^c^	720 (day 14)	-Inadequate MQ exposure
								-Predicted by increased in *pfmdr1* copy number
Adequate MQ exposure alone (n=5)	6	3.55	Reduced (3.0±0.06)	Resistant (45.3±3.1)	>1	320^d^	324 (day 27)	-Possible AS resistant + Inadequate DHA exposure
								-MQ resistant
								-Predicted by increased in *pfmdr1* copy number
	7	3.39	Reduced (3.1±0.05)	Resistant (70.3±6.3)	>1	208^d^	212 (day 28)	-Possible AS resistant + Inadequate DHA exposure
								-MQ resistant
								-Predicted by increased in *pfmdr1* copy number
	8	3.56	Reduced (4.2±0.07)	Resistant (50.0±4.2)	>1	309^d^	489 (day 21)	-Possible AS resistant + Inadequate DHA exposure
								-Mefloquine resistant
								-Predicted by increased in *pfmdr1* copy number
	9	3.64	Sensitive (1.8±0.30)	Resistant (55.3±4.4)	>1	378^d^	510 (day 28)	- Inadequate DHA concentration
								-MQ resistant
								-Predicted by *pfmdr1* copy number
	10	3.22	Sensitive (1.4)	Resistant (35.0±2.9)	>1	489^d^	402 (day 21)	- Inadequate DHA exposure
								-MQ resistant
								-Predicted by *pfmdr1* copy number
Adequate DHA and MQ exposure (n=7)	11	3.97	Reduced (2.9±0.01)	Resistant (118.4±6.1)	>1	-	1,250 (day 17)	-Possible AS resistant
								-MQ resistant,
								-Predicted by increased in *Pfmdr1* copy number
	12	3.45	Reduced (3.7±0.32)	Resistant (95.8±5.4)	>1	-	578 (day 14)	-Possible AS resistant
								-MQ resistant
								-Predicted by increased in *Pfmdr1* copy number
	13	3.33	Sensitive (1.7±0.16)	Resistant (28.8±5.5)	>1	-	102 (day 35)	-MQ resistant
								-Predicted by *pfmdr1* copy number
	14	3.56	Sensitive (1.9±0.10)	Resistant (60.8±7.1)	>1	-	620 (day 14)	-MQ resistant
								-Predicted by *pfmdr1* copy number
	15	3.65	Reduced (3.7±0.21)	Resistant (25.5±0.2)	1	-	610 (day 24)	-Possible AS resistant
								-MQ resistant
	**16**	**3.56**	**Reduced (5.2±0.85)**	**Sensitive (16.6**±0.8	**1**	**-**	**107 (day 35)**	**-Possible AS resistant**
	17	3.44	Sensitive (0.9±0.08)	Sensitive (16.5±2.4)	1	-	67 (day 42)	-Other host-related factors

^a^*Delayed parasite slope half-life if >2.99 h.*

^b^*Mean+SD of three experiments, triplicate each.*

^c^*MQ AUC*_*0-7d*_*which was lower than the lower limit of 95% CI (8.48 mg.day/ml).*

^d^*DHA AUC*_*0-24h*_*which was lower than the lower limit of 95% CI (8.48 ng.h/ml).*

^e^*Day of recrudescence.*

Based on the previously defined criteria, clinical recrudescence response with *in vitro* resistance and/or reduced intrinsic parasitocidal activity of mefloquine and/or artesunate without pharmacokinetic or other host-related factors were confirmed in six cases, two (Nos 13, and 14) with resistance to mefloquine alone, one (No 16) with reduced sensitivity to artesunate alone, and three (Nos 11, 12 and 15) with resistance to mefloquine combined with reduced sensitivity to artesunate. Clinical recrudescence response with *in vitro* resistance and/or reduced intrinsic paratocidal activity of mefloquine/artesunate, together with contribution of pharmacokinetic factor of artesunate and/or mefloquine were confirmed in seven cases: two (Nos 9 and 10) with resistance to mefloquine alone, and five (Nos 1, 2, 6, 7 and 8) with resistance to mefloquine combined with reduced sensitivity to artesunate. Pharmacokinetic factor alone contributed to recrudescence in three cases (Nos 3, 4, and 5), all of which had inadequate mefloquine AUC_0-7 days_. Other host-related factors contributed to recrudescence in one case (No. 17) (Table 1).

*Pfmdr1* copy number data were available in all of the 17 of 29 PCR-confirmed recrudescence cases with complete information on clinico-parasitological response, systemic drug exposure during acute phase and *in vitro* parasite sensitivity. Twelve of 17 (70.1%) isolates in the group with recrudescence response respectively carried >1 *pfmdr1* copy number. Increase in *pfmdr1* copy number was associated with reduced/resistance to mefloquine alone, and artesunate together with mefloquine in 11 cases (four cases, respectively) (Table 1).

### Analysis of contribution of *in vitro* parasite sensitivity and systemic drug exposure and relationship with *pfmdr1* copy number in patients with sensitive response

Analysis of the contribution of *in vitro* parasite sensitivity and systemic drug exposure including relationship with *pfmdr1* copy number in the group with sensitive response was evaluable in 21 of 69 cases (Table 2). Clinical sensitive response with *in vitro* resistance and/or reduced intrinsic parasitocidal activity of mefloquine and/or artesunate without pharmacokinetic factors was confirmed in four cases: one (No 35) with resistance to mefloquine alone, and three (Nos 31, 32 and 33) with resistance to mefloquine combined with reduced sensitivity to artesunate. Clinical sensitive response with *in vitro* resistance and/or reduced intrinsic parasitocidal activity of mefloquine/artesunate, together with contribution of pharmacokinetic factor of mefloquine and/or artesunate were confirmed in five cases: three (Nos 20, 25 and 27) with resistance to mefloquine alone, and two (Nos 18 and 22) with resistance to mefloquine combined with reduced sensitivity to artesunate. Inadequacy of systemic drug exposure was found in 13 cases: four with inadequate mefloquine exposure alone (Nos 18, 19, 20, and 21), nine with inadequate dihydroartemisinin exposure alone (Nos 22, 23, 24, 25, 26, 27, 28, 29 and 30), and two with inadequate exposure of both mefloquine and dihydroartemisinin (Nos 37 and 38).

**Table 2:** **Analysis of contribution of *in vitro *parasite sensitivity and systemic drug exposure during acute phase of infection and relationship with *pfmdr1 *copy number in patients with sensitive response following treatment with a three-day artesunate (AS)-mefloquine (MQ) combination regimen**

**Drug concentrations**	**Case No.**	**Parasite slope half-life (h)**^**a**^	***In vitro *sensitivity (IC**_**50**_**, nM)**^**b**^		***Pfmdr1 *copy number**	**MQ AUC**_**0-7d**_**/ DHA AUC**_**0-24h**_**/**	**Conclusion on parasite sensitivity and adequacy of systemic drung exposure**
			**AS**	**MQ**			
Adequate DHA exposure alone (n= 4)	18	2.90	Reduced (2.4±1.0)	Resistant (34.5±3.2)	>1	8.40^c^	-Possible AS resistant
							-MQ resistant + Inadequate MQ exposure
	19	2.97	Sensitive (1.1±0.15)	Sensitive (8.0±1.4)	1	6.78^c^	-AS sensitive
							-MQ sensitive + Inadequate MQ exposure
							-Predicted by *pfmdr1* copy number
	20	3.01	Sensitive (2.0±0.25)	Resistant (31.3±1.6)	1	7.77^c^	- AS sensitive
							- MQ resistant + Inadequate MQ exposure
							-Predicted by *pfmdr1* copy number
	21	2.78	Sensitive (1.8±0.13)	Sensitive (17.5±3.0)	1	7.09^c^	-AS sensitive
							- MQ sensitive + Inadequate MQ exposure
							-Predicted by *pfmdr1* copy number
Adequate MQ exposure alone (n=9)	22	3.11	Reduced (3.2±0.15)	Resistant (90.0±16.5)	1	402^d^	-Possible AS resistant + Inadequate DHA exposure
							-MQ resistant
							-Predicted by *pfmdr1* copy number
	23	3.23	Sensitive (0.4±0.04)	Sensitive (14.6±2.2)	1	389^d^	-AS sensitive + Inadequate DHA exposure
							-MQ sensitive
							-Predicted by *pfmdr1* copy number
	24	2.78	Sensitive (0.3±0.10)	Sensitive (13.7±2.4)	1	390^d^	-AS sensitive+ Inadequate DHA exposure
							-MQ sensitive
							-Predicted by *pfmdr1* copy number
	25	3.01	Sensitive (1.8±0.31)	Resistant (47.5±1.7)	>1	391^d^	-AS sensitive + Inadequate DHA exposure
							-MQ resistant
	26	2.89	Sensitive (1.6±0.30)	Sensitive (20.9±0.5)	1	398^d^	-AS sensitive+ Inadequate DHA exposure
							-MQ sensitive
							-Predicted by *pfmdr1* copy number entration
	27	2.77	Sensitive (1.6±0.24)	Resistant (27.1±1.0)	1	450^d^	-Possible AS resistant + Inadequate DHA exposure
							-MQ sensitive
							-Predicted by *pfmdr1* copy number
	28	3.01	Sensitive (1.9±0.35)	Sensitive (18.5±2.4)	1	409^d^	-AS sensitive + Inadequate DHA exposure
							-MQ sensitive
							-Predicted by *pfmdr1* copy number
	29	2.89	Sensitive (1.0±0.03)	Sensitive (8.1±0.1)	1	379^d^	-AS sensitive + Inadequate DHA exposure
							-MQ sensitive
							-Predicted by *pfmdr1* copy number
	30	3.01	Sensitive (2.2±0.16)	Sensitive (19.1±1.0)	1	423^d^	-AS sensitive + Inadequate DHA exposure
							-MQ sensitive
							-Predicted by *pfmdr1* copy number
Adequate DHA and MQ exposure (n=6)	31	2.67	Reduced (3.4±0.44)	Resistant (100.7±16.8)	>1	-	-Possible AS resistant
							-MQ resistant
	32	2.89	Reduced (3.8±0.62)	Resistant (62.2±4.6)	>1	-	-Possible AS resistant
							-MQ resistant
	33	3.00	Reduced (3.3±0.37)	Resistant (60.0±4.2)	>1	-	-Possible AS resistant
							-MQ resistant
	34	3.00	Sensitive (1.4±0.31)	Sensitive (6.8±0.5)	1	-	-AS sensitive
							-MQ sensitive
							-Predicted by *pfmdr1* copy number
	35	2.09	Sensitive (0.9±0.13)	Resistant (25.1±0.2)	1	-	-AS sensitive
							-MQ resistant
							-Predicted by *pfmdr1* copy number
	36	2.89	Sensitive (1.6±0.19)	Sensitive (21.2±0.3)	1	-	-AS sensitive
							-MQ sensitive
							-Predicted by *pfmdr1* copy number
Inadequate DHA and MQ exposure (n=2)	37	2.90	Sensitive (1.5±0.23)	Sensitive (14.3±2.7)	>1	5.34^c^	-AS sensitive
						234^d^	-MQ sensitive
	38	3.01	Sensitive (1.9±0.28)	Sensitive (22.1±0.4)	1	7.09^c^	-AS sensitive
						209^d^	-MQ sensitive
							-Predicted by *pfmdr1* copy number

^a^*Delayed parasite slope half-life if > 2.99 h.*

^*b*^*Mean ± SD of 3 experiments, triplicate each.*

^c^*MQ AUC*_*0-7d*_*which was lower than the lower limit of 95% CI (8.48 μg.day/ml).*

^d^*DHA AUC*_*0-24h*_*which was lower than the lower limit of 95% CI (8.48 ng.h/ml).*

*Pfmdr1* copy number data were available in all of the 21 cases with complete information on clinico-parasitological response, drug concentrations and *in vitro* parasite sensitivity. Fifteen of 21 (71.4%) carried only one *pfmdr1* copy number (Table 2).

### Association between treatment response and systemic drug exposure

No significant association between mefloquine and dihydroartemisinin exposure (AUC_0-7 days_ and AUC_0-24h_) and treatment response was found (*p* = 0.137 and 0.583, respectively).

## Discussion

Accumulating evidence suggests a decline in the efficacy and some degree of resistance of *P. falciparum* in the Greater Maekong Subregion (GMS) to artemisinins. Early evidence came from western Cambodia and the Thai-Cambodian border in patients following treatment with either artesunate monotherapy or artesunate-mefloquine [2,3,9,10]. Although results of the containment project in seven provinces of Thailand bordering Cambodia (Buriram, Chantaburi, Sakaew, Srisaket, Surin, and Trat) during 2009–2011, in a total of 1,709 *P. falciparum*-positive cases, suggest that the therapeutic efficacy of artesunate-mefloquine remains at an acceptable level with cure rate of greater than 90%, continuous monitoring of *P. falciparum* resistance in both border areas is critical [29]. With regard to the Thai-Myanmar border, until recently [11,12], there has been no clear evidence of a significant reduction in artemisinin efficacy at either clinical or *in vitro* sensitivity level. A longitudinal investigation in a total of 3,202 patients during 2001–2010 from malaria clinics, Shoklo Malaria Research Unit, Tak Province, unveiled that genetically determined artemisinin resistance in *P. falciparum* may have emerged along the Thai-Myanmar border at least eight years ago and has since markedly increased [12]. The clinical efficacy of artesunate-mefloquine combination therapy is beginning to decline in the Thai-Myanmar border and resistance is not only confined to western Cambodia and areas along the Thai-Cambodian border. Due to the limitation of the study design however, it was not possible to attribute treatment failures in these studies to resistance or host-related factors (e g, pharmacokinetics). Furthermore, if resistance actually occurred, it was unclear whether this was due to intrinsic parasite resistance to artesunate alone, mefloquine alone, or both, because of the pre-existing background of mefloquine resistance in these areas. In order to exclude the contribution of host and confounding factors from the partner drug mefloquine, a series of investigation with artesunate monotherapy (2–6 mg/kg body weight/day for seven days) was performed during 2006–2008 using stringent criteria for defining artemisinin resistance with integrated *in vivo-in vitro* approach [4-8].

The present study was designed to identify the treatment failure cases due to intrinsic parasite factor to each component of the three-day regimen. Resistance or decline in parasite susceptibility was confirmed based on integrated information on clinicopathological assessment together with *in vitro* sensitivity (intrinsic parasite resistance) and systemic drug exposure (pharmacokinetic factor) in 17 out of 29 patients with recrudescence (LPF) following treatment with a three-day combination regimen of artesunate-mefloquine [11]. All had significant delay in parasite clearance rate (slope half-life) compared with the sensitive cases. Despite the fact that the delay in parasite clearance is influenced by host-related factors, it is proposed as a sensitive marker of reduced susceptibility of artemisinin component of the ACT than recrudescence rate [30]. The main effect of artemisinins is proposed to be on the slope of the log-linear decline in parasite clearance and thus, the slope half-life [15].

Resistance of the *P. falciparum* isolates to mefloquine or aretsunate was defined according to *in vitro* sensitivity criteria. Existing data on the relationship between *in vitro* sensitivity and artemisinin are controversial. Although lack of significant correlation between *in vitro* sensitivity of artemisinins and clinical response was found in some studies [4,31], good correlation was observed in most studies [2,3,9,26,32,33]. Adequacy of mefloquine and dihydroartemisinin systemic drug exposure was defined based on the lower limits of 95% CI of the median AUC_0-7 days_ and AUC_0-24h_, respectively, in the sensitive group. Based on this defined criteria, results suggest that low level of a decline in sensitivity of *P. falciparum* to artesunate (in terms of a small increase in IC_50_ of artesunate and number of identified cases with reduced sensitivity to artesunate) exists in this area on a background of pre-existing mefloquine resistance, and both parasite in conjunction with host-related factors significantly contributed to high failure rate in this group of patients. There was only one (out of 17) confirmed case with reduced sensitivity to artesunate alone, while there were three cases with reduced sensitivity/resistance to both artesunate and mefloquine. Pharmacokinetic factor contributed to about 58.8% (10 of 17 cases) of the total recrudescence cases. Inadequacy of mefloquine and dihydrortemisinin systemic drug exposure was observed in five and five cases, respectively. However, there was no significant difference in the systemic exposure of both mefloquine and dihydroartemisinin (AUC_0-7 days_ and AUC_0-24h_) in patients with treatment failure compared with those with sensitive response.

Host-related factor contributing to treatment failure in one case could not be definitely defined. It is noted that the *in vitro* sensitivity of the only one isolate with identified resistance/reduced sensitivity to artesunate alone (No 16) was markedly low compared with others (IC_50_ 5.2 nM). Furthermore, in one (No 11) of the three cases with *in vitro* resistance/reduced sensitivity to both mefloquine and artesunate, mefloquine concentration of as high as 1,250 ng/ml was detected on the day of recrudescence (day 17). It is of note that this high level was still inadequate to completely eliminate residual parasites on background of resistance/reduced sensitivity to both mefloquine and artesunate. In the other two cases with contribution of mefloquine pharmacokinetic factor (Nos 1 and 2), variable whole blood mefloquine concentration on the days of recrudescence of 610 and 100 ng/ml was observed. The relatively low drug concentrations in some of the recrudescence cases could be due to variability in pharmacokinetics of mefloquine and artesunate/dihydroartemisinin, and these concentrations were no longer adequate once the level of resistance to either mefloquine or artesunate was aggravated. Systemic exposure of both drugs during the acute phase infection was used as a criterion to define adequacy of drug levels. Whole blood mefloquine concentration on day 1 of treatment has been reported to be an important determinant of successful treatment [34], but there has been no defined threshold levels of artesunate/dihydroartemisinin for treatment of falciparum malaria. Sensitivity of *P. falciparum* isolates in this area to mefloquine was still at the resistance level (43.3% of the isolates with recrudescence) after a certain period of improvement [27]. Mefloquine was used as monotherapeutic treatment of acute uncomplicated falciparum in this area long before the introduction of the combination regimen and thus mefloquine resistance had already reached a level too extreme to protect the development and spread of artesunate resistance. The sensitivity of *P. falciparum* to artemisinins would be compromised by intensifying resistance to mefloquine. Decline of *in vitro* sensitivity of parasite in this, as well as other areas to artesunate has been demonstrated [4,7,35] and it is noted for a ten-fold decrease in *in vitro* artemisinin sensitivity observed over a 10-year period in north-western Thailand [36]. In a previous study, decreased *in vitro* susceptibility to dihydroartemisinin (IC_50_ 21.2 nM) and artesunate (16.3 nM) was reported in a patient returning from south of Laos and north of Thailand [26]. In view of the short half-life of oral artesunate on the other hand, it is expected that the drug exerts little drug pressure, provided the treatment generally results in parasitological cure. Compared with western Cambodia and areas along the Thai-Cambodian border, intensity of malaria transmission is relatively low in the north-western border areas of Thailand. This would lead to lower selective drug pressure and drug resistance. Although artemisinin resistance starts to gradually emerge [37,38], relatively good parasitologic responses to artemisinins are observed in this border area even after almost 20 years of intensive use [31].

Analysis of the contribution of *in vitro* parasite sensitivity and drug concentrations and relationship with *pfmdr1* copy number in the group with sensitive response was performed in 21 of 69 cases. Results indicate contribution of both parasite and pharmacokinetic factors in treatment response, but with different magnitude. Pharmacokinetic factor contributed to 71.4% (15 of 21 cases) of variation in mefloquine and/or dihydroartemisinin concentrations in this group of patients, of which 35.3% (six of 17 cases) were due to variable mefloquine pharmacokinetics (alone or together with dihydroartemisinin) and 64.7% (11 of 17 cases) were due to variable artesunate/dihydroartemisinin pharmacokinetics (alone or together with mefloquine). *In vitro* resistance/reduced sensitivity to mefloquine and aresunate was found in 36.7% (nine of 17 cases) and 43.3% (12 of 17 cases) of all cases, respecrtively. In the group with recrudescence response, pharmacokinetic factor contributed to 58.8% (ten of 17 cases) of variation in melfoquine and/or dihydroartemisinin concentrations; 50% (five of ten cases) were due to variable mefloquine pharmacokinetics (alone or together with dihydroartemisinin) and 50% (five of ten cases) were due to variable artesunate/dihydroartemisinin pharmacokinetics (alone or together with mefloquine). Resistance/reduced sensitivity to mefloquine and aresunate was found in 23.8% (five of 21 cases) and 42.9% (nine of 21 cases) of all cases, respectively. Altogether, these findings may suggest that the influence of pharmacokinetic variability of mefloquine influences more on treatment response than of artesunate/dihydroartemisinin on background of mefloquine resistance together with decreasing susceptibility of parasite to artesunate. Although statistical significance could not be achieved due to a marked variability, the IC_50_ of both artesunate (medians of 49.9 *vs* 21.1 nM) and mefloquine (medians of 2.8 *vs* 1.8 nM) tended to be higher in isolates obtained from patients with recrudescence response. In the two cases with sensitive response (Nos 18 and 20), sensitivity of the parasite to both artesunate and mefloquine is relatively high and even with low systemic exposure of mefloquine, were still adequate to completely eliminate all the parasites. In the other three cases (Nos 31, 32 and 33) with *in vitro* resistance/reduced sensitivity to mefloquine and artesunate, radical cure could be obtained as far as adequate systemic exposure of both mefloquine and dihydroartemisinin were achieved.

A consensus for the molecular and cellular mechanisms of artemisinin resistance has not emerged. Despite continuous efforts to uncover definite molecular markers for resistance to artemisinins in *P. falciparum,* no valid marker has been identified and confirmed, which precludes efficient monitoring of emerging and spreading resistance. Results from various molecular studies varied, probably reflecting a multigenetic nature of artemisinin resistance. Among these, polymorphisms of PfMDR1 (an ATP-binding cassette (ABC) transporter residing on the digestive vacuolar membrane of the parasite, where it transports solutes, including anti-malarial drugs into the digestive vacuole) encoded by the *pfmdr1* gene have received the most attention. Several mutations (N86Y, Y184F, S1034C, N1042D and D1246Y) and copy number variation that occurred in *pfmdr1* from field isolates collected from several endemic areas are associated with altered sensitivity to artemisinins and their combination partner mefloquine [39-42]. Amplification of PfMDR1 copy number has been proposed as a key determinant for both *in vivo* and *in vitro* resistance to both artemisinins and mefloquine in Cambodia and areas along the Thai-Cambodian and Thai-Myanmar borders [26,35,43-47]. Nevertheless, some clinical trials could not confirm the link between PfMDR1 polymorphism/copy variation and artemisinin resistance [4-8]. There appeared to be no association between the mutation or amplification of *pfatp6*, the sarco/endoplasmic reticulum Ca^2+^-ATPase, in *P. falciparum* isolates in Thailand [38]. In this study, increased copy number of *pfmdr1* was shown to be associated with treatment failure and a decline in *in vitro* susceptibility of *P. falciparum* isolates in this area following artesunate-mefloquine combination, with approximately 40% of the isolates carrying *pfmdr1* copy number between two and six copies. *Pfmdr1* copy number correlated well with clinical treatment response in both groups of patients with sensitive and recrudescence response. Approximately 70.1% (12 of 17 cases) and 71.4% (15 of 21 cases) in the groups with sensitive and recrudescence response carried gene copy number >1 (two to six) and one, respectively. Resistance to ACT may evolve even when the two drugs within the combination are taken simultaneously and amplification of the *pfmdr1* gene may partly contribute to this phenotype.

## Conclusions

Despite the evidence of a low level of a decline in sensitivity of *P. falciparum* isolates to artemisinins in areas along the Thai-Myanmar border, ACT would be expected to remain the key anti-malarial drug for treatment of multidrug resistance *P. falciparum*. Continued monitoring and active surveillance of clinical efficacy of ACT including identification of true artemisinin resistant parasite is required for appropriate implementation of malaria control policy in this area.

## Competing interests

The authors have declared that they have no competing interests.

## Authors’ contributions

KN and JK conceived and designed the experiments. PM, WC and RR performed the experiments. PM and WC analysed the data. KN and JK wrote the paper. All authors read and approved the final manuscript.
